# Impact of Assistant Experience on Perioperative Outcomes of Simple and Radical Laparoscopic Nephrectomy: Does It Really Matter?

**DOI:** 10.3390/medicina60010045

**Published:** 2023-12-26

**Authors:** Francesca Ambrosini, Guglielmo Mantica, Giovanni Marchi, Enrico Vecchio, Benedetta Col, Luca Gaia Genova, Giorgia Trani, Arianna Ferrari, Carlo Terrone

**Affiliations:** 1 IRCCS Ospedale Policlinico San Martino, 16132 Genova, Italy; f.ambrosini1@gmail.com (F.A.); giovanni.marchi5@studio.unibo.it (G.M.); enrico.vecchio.5@gmail.com (E.V.); col.benedetta@gmail.com (B.C.); lucagenovagaia@gmail.com (L.G.G.); gtrani57@gmail.com (G.T.); ariannaf1996@gmail.com (A.F.); carlo.terrone@med.uniupo.it (C.T.); 2 Department of Surgical and Diagnostic Integrated Sciences (DISC), University of Genova, 16131 Genova, Italy

**Keywords:** minimally invasive nephrectomy, laparoscopic surgery, assistant experience, operative outcomes, surgical training

## Abstract

*Background and Objectives*: While systematic reviews highlight the advantages of laparoscopic nephrectomy over traditional open surgery, the impact of an assistant’s experience on surgical outcomes remains unclear. This study aims to evaluate whether the level of assistant expertise influences laparoscopic nephrectomy outcomes. *Materials and Methods*: Retrospective data from our institutional database were analyzed for patients who underwent laparoscopic nephrectomy between January 2018 and December 2022. Procedures were performed by a highly experienced surgeon, including postgraduate year (PGY)-3 to PGY-5 residents as assistants. Senior-level assistants had completed at least 10 procedures. Patient characteristics, surgical outcomes, and postoperative details were collected. Multivariable linear and logistic regression models were performed to test the effect of assistant experience (low vs. high) on estimated blood loss (EBL), length of stay (LOS), operative time (OT), and postoperative complications. *Results*: 105 patients were included, where 53% had highly experienced assistants and 47% had less experienced ones. Low assistant experience and higher BMI predicted longer operative time (OT), confirmed by multivariable regression (β = 40.5, confidence interval [CI] 18.7–62.3, *p* < 0.001). Assistant experience did not significantly affect EBL or LOS after adjusting for covariates (β = −14.2, CI −91.8–63.3, *p* = 0.7 and β = −0.83, CI −2.7–1.02, *p* = 0.4, respectively). There was no correlation between assistant experience and postoperative complications. *Conclusions*: Assistant experience does not significantly impact complications, EBL, and LOS in laparoscopic nephrectomy. Surgeries with less experienced assistants had longer OT, but the overall clinical impact seems limited. Trainee involvement remains safe, guided by experienced surgeons.

## 1. Introduction

During the last decades, minimally invasive nephrectomy has slowly gained popularity due to its benefits over traditional open surgery [1,2]. Several systematic reviews and meta-analyses assessing the surgical and oncologic outcomes of laparoscopic nephrectomy have consistently demonstrated that this approach provides perioperative advantages over open radical nephrectomy (ORN), including reduced length of hospital stay, lower estimated blood loss, and decreased transfusion rates, while achieving comparable oncologic outcomes [1,2].

While the integration of laparoscopy and robotics into surgical practice have introduced positive results for patients, they also bring a learning curve, which has been investigated in different studies [3].

Laparoscopic nephrectomy is one of the most commonly performed laparoscopic procedures in which residents in urology can be involved [4]. While the role of surgical experience on patient outcomes in the context of nephron-sparing surgery has been widely explored [5,6,7,8], there is still a significant gap in knowledge regarding the impact of resident training on the surgical outcomes of radical nephrectomy (RN).

The potential risks associated with having less experienced assistants in the operating room, such as longer operative times [7,8], negative impacts on patient outcomes [9,10,11], and increased risk of surgical complications [11], highlight the importance of understanding the dynamics of resident involvement in laparoscopic nephrectomy. Evidence of the actual impact of resident expertise on surgical outcomes of laparoscopic radical or simple nephrectomy is lacking in the literature.

To address this issue, we retrospectively evaluated whether the level of experience of the assistant had significant influence on operative outcomes of laparoscopic nephrectomy.

## 2. Materials and Methods

### 2.1. Study Population and Outcomes

We retrospectively analyzed data prospectively recorded in our institutional database of consecutive patients who underwent simple or radical laparoscopic nephrectomy at our institution between January 2018 and December 2022. Simple nephrectomy was performed in the case of renal obstruction, recurrent infection, or renal stones that resulted in a poorly- or non-functioning kidney. Radical nephrectomy was performed for cT2 renal tumours or cT1 renal masses when partial nephrectomy was not considered technically safe and feasible according to the European Association of Urology Guidelines. All procedures were performed by a single highly experienced surgeon (C.T.) who, at baseline, had completed more than 500 minimally invasive renal procedures (partial and radical/simple nephrectomies). In all cases, either a postgraduate year (PGY)-3, PGY-4, or PGY-5 resident was involved as an assistant. A senior-level assistant was defined as someone who had performed at least 10 laparoscopic nephrectomies and was actively involved as the scrubbed assistant during the procedures. The residents were always supervised by an experienced surgeon. All consecutive patients who underwent surgery (laparoscopic nephrectomy) during the study period were included in the study. Patients with missing data regarding the experience of the assistant were excluded. Only laparoscopic nephrectomies carried out with a transperitoneal approach were included, while retroperitoneal were excluded from the study. Patient characteristics were assessed for age, gender, body mass index (BMI), surgical indication and laterality. Postoperative outcomes included operative time (OT), estimated blood loss (EBL), postoperative complications scored according to the Clavien-Dindo classification [9], and length of hospital stay (LOS).

### 2.2. Surgical Technique

The surgical procedures were performed using a transperitoneal approach. The patient was placed in a 70-degree semi-lateral decubitus position. Using the Hasson technique, the pneumoperitoneum was reached with a pressure of 12 mmHg. A 10 mm camera trocar was placed superior and lateral to the umbilicus and lateral to the rectus muscle to avoid the inferior epigastric vessels; subsequently a 12 mm upper and a 12 or 5 mm lower quadrant trocar were placed. In the case of a right nephrectomy, a 5 mm trocar can optionally be placed subxiphoidally to support liver retraction.

After complete mobilization of the colon by incising the white line of Toldt, the plane between the mesentery of the colon and the anterior surface of Gerota fascia is identified. Dissection advanced medially, gradually revealing the hilum and allowing isolation and mobilization of the renal vein. This maneuver usually provided access to the artery. The renal artery was exposed and clipped with Hem-o-lok clips and finally transected with blunt scissors. The renal vein was divided using Hem-o-lok clips.

Once the renal vessels are completely severed, complete mobilization of the entire kidney is performed, including the fat capsule and, if necessary, the adrenal gland. Before the ureter was transected, it was ligated with Hem-o-lok clips.

The specimen was entrapped within an ENDO-bag, which was subsequently extracted intact through a 5 cm incision made by splitting the flank muscles.

### 2.3. Training Program at Our Institution

Training in laparoscopic nephrectomy at our center has been standardized for about ten years, with progressive changes and adaptations of the training program based on different factors such as the technological innovations and adopted techniques. This teaching begins during the second year of urology residency (PGY-2). The first step is characterized by frontal lessons on kidney surgical anatomy and on the basic principles of laparoscopy. During this module, the trainee approaching laparoscopic surgery learns the theoretical and anatomical steps of laparoscopic nephrectomy and the various materials and devices used during the surgical procedure itself. The residents are shown different videos of nephrectomies carried out by the different surgeons operating at the institute, and in particular they are also shown videos about any surgical situations of doubt and difficulty which the first operator may find themself in during the procedure itself, for example: damage to the renal arteries and veins, excessive bleeding, etc. In the next module, residents become part of the operating team for at least 5 procedures. They will be physically present on the operating field as active spectators of the surgical operation, in order to fully learn the operation steps and how to use and run the different devices. They are invited to view both the operation itself from the point of view of the first operator, but also from the point of view of the assistant surgeon. In the next module, which is theoretical, the new residents are taught by senior residents (PGY-3, PGY-4, PGY-5), again using video support from previous interventions. During this step, they are focused on the role of the assistant surgeon during the laparoscopic nephrectomy, showing them what and how the assistant must do in the different surgical steps to effectively help the first surgeon. Next, residents will perform their first assistant-assisted laparoscopic nephrectomy procedures. During the first two–three interventions, a senior resident will be present as support, in case of difficulties. In subsequent operations, the trainees will be able to independently carry out the role of assistant surgeon. The training continues in the following two years with the aim of becoming a first surgeon and mastering a standard laparoscopic nephrectomy procedure. As a first operator, the training becomes modular, in 5 steps consisting of 10 cases each. In the first 10 cases the trainees position the patient on the operating bed and introduce the trocars. In the next step, they actively mobilize the colon by incising the white line of Toldt and expose the Gerota’s fascia. Subsequently, they perform the dissection of the hilum allowing for the isolation and exposition of the renal vessels. In the following step they perform the division and transection of the renal artery and vein. They also perform the transection and ligation of the ureter. Finally, they perform the mobilization of the entire kidney, including the fat capsule and, if necessary, the adrenal gland. They also perform the rescue of the final specimen. The nephrectomy training modular program ends when, as senior resident, the trainees are able to perform their first laparoscopic nephrectomy under the supervision of a consultant.

### 2.4. Impact of the SARS-CoV-2 Pandemic on the Training Model

Part of the study was carried out during the period characterized by the global SARS-CoV-2 pandemic which officially affected our country since the 9 March 2020 [12]. During this period, the surgical activity at our center did not stop. However, surgical case volume was significantly reduced for at least 6 months. This reduction in surgical activity consequently also led to a necessary continuous adaptation of the training model used, since the reduction in the overall number of operations led to the necessary and temporary reduction in the number of cases per surgical step. During this period, academic tutors attempted to overcome the reduction in clinical practice by increasing the number of lectures. Through the use of video support, even greater attention was paid to the phases of the operation itself, the possible complications and critical situations during the operation.

### 2.5. Statistical Analysis

Descriptive statistics included frequencies and proportions for categorical variables. Means, medians, and interquartile ranges (IQR) were reported for continuously coded variables. The χ^2^ tested the statistical significance in proportional differences. The *t*-test, the Kruskal—Wallis rank sum test, Fisher’s exact test, and Pearson’s Chi-squared test examined the statistical significance of means and median differences. We relied on univariate and multivariate analysis including uni- and multivariable linear and logistic regression models, after adjustment for covariates (BMI, age, and side), to test the effect of the assistant experience (low vs. high) on EBL, LOS, and OT (all continuously coded), and postoperative complications (yes vs. no). Added-variable plots were used to visualize the results of multivariable regression models. The R software environment for statistical computing and graphics (version 4.1.2) was used for all statistical analyses. All tests were two-sided with a level of significance set at *p* < 0.05.

## 3. Results

Descriptive characteristics of the study cohort, stratified by bedside assistant experience, are shown in Table 1.

Overall, data from 105 patients operated on at our institution were included in the study of whom 20 (19%) underwent simple nephrectomy and 85 (81%) underwent radical nephrectomy. The median size of renal cancer was 6 cm (interquartile range [IQR] 5–8.5 cm]) (Table 1).

A total of 56 (53%) cases underwent surgery performed by a highly experienced assistant, while 49 (47%) by an inexperienced assistant.

Among the patients in the “highly experienced assistant” group, 3 experienced Clavien-Dindo 3a complications (1 cardiovascular complication, 1 retroperitoneal abscess requiring surgical drainage, and 1 bleeding requiring embolization performed by interventional radiologists), 1 had Clavien-Dindo 3b complication (bleeding requiring surgery), and 2 had Clavien-Dindo 4 complications (1 intraoperative diaphragmatic injury and 1 pulmonary complication requiring postoperative intensive-care management), respectively.

Among patients who underwent surgery with a low-experience assistant, 1 reported a Clavien-Dindo 4 complication (1 intraoperative bleeding due to spleen injury requiring postoperative intensive-care management).

There were no variations or deviations from the standard laparoscopic nephrectomy procedure. The cases reported are a single surgeon’s series and the surgical technique used remained the same throughout the study period.

The results of the regression analysis are reported in Table 2 and Table 3.

**Figure 1:**
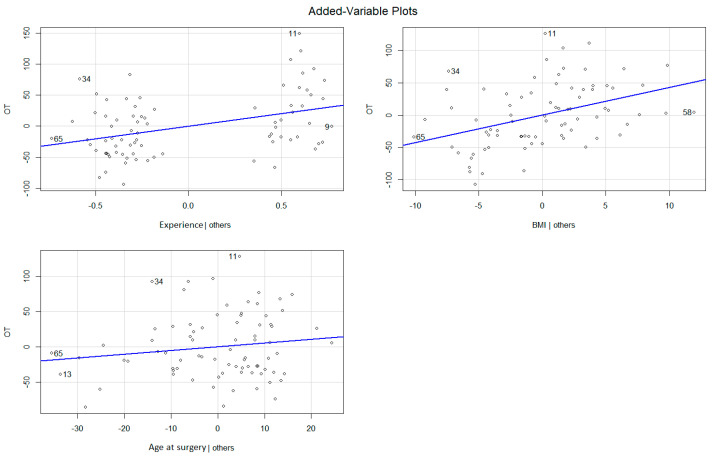
Added-variable plot showing the results of multivariable regression model for the operative time.

The two groups were comparable according to age at surgery, BMI, gender, and surgical laterality. According to univariable linear regression models, low assistant experience and BMI were found to be predictors of longer OT (*p* = 0.04 and *p* = 0.006, respectively). Low assistant experience and BMI retained the predictor status for OT also in the multivariable linear regression model adjusted for age at surgery (β = 40.5, confidence interval [CI] 18.7–62.3; β = 4.3, CI 2.04–6.5; both *p* < 0.001) (Table 2, Figure 1). In the multivariable linear regression models, adjusted to age at surgery, BMI and laterality, assistant’s experience had no effect neither on the EBL nor on the LOS (β = −14.2, CI −91.8–63.3, *p* = 0.7 and β = −0.83, CI −2.7–1.02, *p* = 0.4, respectively). Both the uni- and multivariable logistic regression models showed no correlation between assistant’s experience and the rate of postoperative complications (OR 0.5, *p* = 0.1 and OR 0.6, *p* = 0.4, respectively) (Table 2 and Table 3). BMI, age at surgery and laterality were not predictors of postoperative complications and LOS in both uni- and multivariable models (Table 2 and Table 3). Both BMI and laterality influenced EBL in the multivariable regression models (β = 10.5, CI 2.6–18.4, *p* = 0.01 and β = −85.2, CI = −161.4–9.07, *p* = 0.03, respectively) (Table 2).

## 4. Discussion

“I run on the road, long before I dance under the lights”. This is a famous phrase by Muhammad Ali that represents training. As the boxing champion had to train on the road for years and years before getting to play under the lights of the boxing rings, similarly, young surgeons are expected to undergo a complex and lengthy learning process before becoming a brilliant specialist. This process’s duration can be variable and depends on many factors such as the trainee’s attitudes and skills, the teaching ability of the tutors, the availability of simulators, and training models to reduce learning curves. However, this process collides with an inevitable factor given by the patient, who is the inevitable focus of the healthcare process. In the past, training has always been based on the theme of “see one, do one, teach one” introduced by Sir William Stewart Halsted. However, this simple, fast and practical learning scheme, still in use in some settings, has gradually been abandoned for obvious reasons related to ethics and the safety of the patients themselves.

In recent decades, the topic of training residents in surgical matters and in particular in urology has been under the spotlight [13]. Multiple studies have explored the role and impact of urological residents in different settings and activities: academic, clinical, surgical, and healthcare [14,15]. Furthermore, there are also numerous studies that have investigated the quality of life of the resident and their actual possibility of academic and surgical learning in certain settings and countries [16,17,18]. At the same time, the role of the patient has become even more fundamental, both as the object of treatment and training, and as a passive part of the training of new trainees itself [19].

In this regard, in order to plan, define and organize standardized and reproducible training for urology residents, many urological associations have moved at national and supranational levels, such as the European Association of Urology (EAU) [20]. This company first defined annual courses, organized into modules of different levels, in various urological subdisciplines. Subsequently, it produced urological certifications, such as the European training in basic laparoscopic urological skills (E-BLUS). In detail, the E-BLUS is a program offered to residents and urologists who want to improve basic skills in laparoscopy under the training and supervision of international experts in laparoscopy. The E-BLUS program includes Hands-on Training (HOT) courses of different levels carried out under the guidance of experienced tutors, training-box exercises developed and validated by the Dutch project, Training in Urology (TiU), to train basic skills needed in urological laparoscopy and finally the E-BLUS examination and certification.

In urologic surgical training, the integration of minimally invasive techniques is of great importance, and the involvement of residents is both common and foreseen in high-volume centers. Optimizing resident participation in minimally invasive urologic procedures without compromising patient outcomes is of paramount importance.

Nevertheless, the impact of the assistant’s experience on surgical outcomes of laparoscopic nephrectomy has not been widely explored. To the best of our knowledge, this is the first study evaluating the impact of the expertise of the assistant on the surgical outcomes of laparoscopic radical or simple nephrectomy. Our analysis revealed several noteworthy findings. First, we found that low assistant experience significantly increased the OT.

OT is a critical factor, as it not only affects patient outcomes but also has implications for resource utilization and the efficiency of surgery. While concerns exist regarding the potential drawbacks of increased operative duration, such as increased anesthesia exposure and longer LOS [10], extended OT did not lead to a higher rate of perioperative complications according to our results. Similar data have been reported in studies assessing the impact of the level of assistant experience on other urological minimally invasive surgical procedures.

Mitsinikos E. et al. observed that increased LOS (+1 day) and OT (+16 min) were the only relevant parameters negatively impacted by residents involvement during robot-assisted partial nephrectomy (RAPN) [11]. An increased OT when trainees participate in RAPN was confirmed by Myers A. et al. [7] and Khene Z et al. [8]. According to their results, the rate of complication was not negatively affected despite the surgical time.

Thomas A. et al. analysed 162 robot-assisted radical prostatectomy (RARPs) with trainee involvement and found that surgeries with low-experienced assistants were longer and with higher EBL compared to the procedures performed only by high-experienced surgeons [21].

While increased OT seems to be a consistently reported outcome in literature when a less experienced assistant participates in surgical procedures, it is worth noting that the clinical impact on the patient might be less significant than initially perceived.

Albo et al. corroborated that the learning curve of bed assistants during robot-assisted radical prostatectomy (RARP) did not appear to significantly impact surgical outcomes such as OT, EBL, LOS. Instead, the predominant factor influencing these results was found to be the experience of the surgeon [22].

Secondly, we observed that the involvement of low-experience assistants did not negatively affect EBL and LOS.

These results underscore how the level of assistant experience might not be clinically significant and do not compromise the quality and safety of patients’ outcome.

Our results are consistent with some previous studies addressing the same endpoints for different minimally invasive urological procedures [8]. On the contrary, other analyses suggested a potential negative impact of a fellow on a robot-assisted partial nephrectomy, but the clinical relevance of these differences is questionable [23].

Taking all these points together, our results support the involvement of assistants with a low level of experience in laparoscopic nephrectomy given the surgical procedures are executed with a reasonable degree of safety.

Other factors beyond assistant experience, such as surgical technique, patient characteristics, and surgeon expertise, seem to play a more dominant role in influencing perioperative outcomes [24,25].

Nevertheless, considering the limitations of our study, we believe that the experience of assistants should always be considered in surgical case planning, especially when a senior surgeon is not available as a supervisor.

In our study, there was always an experienced surgeon present in the operating room to provide supervision and support in the case of emergency, in addition to the primary surgeon.

To overcome the potential drawbacks of assistants with low levels of experience during surgeries, training and simulation programs could indeed hold promise in enhancing outcomes [26,27,28,29]. It has been demonstrated that, considering the comparable clinical outcomes between patients treated by inexperienced surgeons and those treated by skilled surgeons, laparoscopic nephrectomy presents a viable opportunity for training purposes [5].

Moreover, in light of technological advancements, innovative training systems have emerged that have proven effective in enhancing education, including virtual reality simulators.

Miyata et al. evaluated the LapVision virtual reality simulator’s laparoscopic radical nephrectomy module, demonstrating its efficacy in assessing surgical skills, especially for integrated laparoscopic training programs, emphasizing its potential for broader adoption in advanced procedure education [30].

Furthermore, despite the complexity of organizations, high-volume centers including structured training programs tend to achieve overall better outcomes extending beyond the assistant’s individual skill development [31,32,33].

Some limitations should be mentioned. Firstly, the relatively limited number of patients included in the study may limit the power of the regression analyses which may suffer from overfitting. The small sample size may affect the reliability and generalizability of our results, which should be interpreted with caution. The descriptive nature of the data urges caution when drawing definitive conclusions. Although the insights gained from our study appear valuable in the context of the available data, a larger and more diverse sample would improve the robustness of our conclusions.

Secondly, we focused on procedures performed by a single, highly experienced surgeon, and this may limit the external validity of the study. In fact, if the impact of an inexperienced assistant may be limited in the case of a first surgeon with great experience in this type of operation, this may not apply to less experienced surgeons or, worse, during the height of their learning curve.

Moreover, the criteria of completing a minimum of 10 procedures to qualify as a senior-level assistant was chosen arbitrarily and might not comprehensively capture an assistant’s “expertise”. The choice of a threshold of 10 procedures took into account several factors, including a balance between a sufficient number of laparoscopic nephrectomy cases and consideration of the practical limitations of residency training programs. We acknowledge that there is no clear and standardized rationale for setting this cut-off. In any case, there is no similar study in the literature. Even with this arbitrarily established threshold, a significant improvement in OT has been demonstrated in cases where surgeries were performed by assistants with this 10-case experience.

A further limitation to our study, as well as to the majority of surgical training case studies published in the reference period, may have been represented by the SARS-CoV-2 pandemic that broke out in the first months of 2020. In fact, this global-scale event, although it did not stop surgical activity at our center (which represents a reference surgical hub for our region and continued to carry out minimally invasive oncological surgery operations throughout the pandemic period), certainly reduced their number, at least in part. This led to a delay in the learning times generally seen by our modular training for laparoscopic nephrectomy, which could, at least in part, have influenced some of the results. However, considering that the significant reduction in surgical cases lasted only about 6 months, this should have had no more than minimal influence on the results of both the training and the study itself.

Another limitation is the fact that, to make the study more homogeneous, we decided to study the effect of the assistant only on laparoscopic nephrectomies with a transperineal approach. This is the most used approach in our center. However, the results obtained may not necessarily also apply to laparoscopic nephrectomy with a retroperitoneal approach, which presents slightly different positioning of the patient, trocars, and surgical steps.

Finally, the retrospective nature of the study inevitably introduced some selection bias that could not be overcome. Specifically, we would like to highlight that the higher rates of Clavien-Dindo grade 3–4 complications and the greater percentage of right nephrectomies in the highly experienced assistant group could potentially be attributed, not only to the casualty, but also to preoperative decision-making processes, such as scheduling specific types of cases to ensure the availability of certain assistants. This aspect should be considered when interpreting the results.

Nevertheless, we believed that this study holds significant relevance as it underscores the relatively marginal role of assistant experience while highlighting the pivotal importance of implementing training programs to enhance outcomes. By safely investing in effective training programs, we pave the way for more skilled surgical teams, ultimately leading to improved patient care in the long term. Of fundamental importance remains the surgical training of residents using laparoscopy simulators, and a precise knowledge of the surgical steps, which should be the basics before assisting in the operatory theater [34].

## 5. Conclusions

Our data showed that assistants with a low level of experience do not appear to have a negative impact on complications, estimated blood loss and length of stay for laparoscopic nephrectomy but increase the operative time.

Trainee involvement in laparoscopic nephrectomy, particularly under the supervision of a highly experienced surgeon, demonstrates comparable outcomes to those achieved with high-experience assistants.

Further research with larger sample sizes and a prospective study design is warranted to provide more robust insights into the impact of assistant experience on surgical outcomes. The gradual participation of trainees in laparoscopic nephrectomy operations is of fundamental importance in order to train surgeons capable of operating and mastering major urological surgery operations safely for patients. The presence in the surgical field of a tutor with high experience in laparoscopic surgery is mandatory both to obtain expected outcomes in operated patients and to best guide the training of new surgeons.

## Figures and Tables

**Table 1:** Descriptive characteristics of 105 patients who underwent simple or radical laparoscopic nephrectomy at a single institution between 2018 and 2022, stratified by bedside assistant’s experience.

Characteristic	High-Experience Assistant (>10 Procedures)	Low-Experience Assistant (≤10 Procedures)	*p*-Value ^1^
Number of patients, n (%)	56 (53%)	49 (47%)	
Age at surgery (yr), median (IQR)	71 (60, 78)	67 (59, 76)	0.4
Gender, n (%)			0.8
Female	24 (43%)	22 (45%)	
Male	32 (57%)	27 (55%)	
BMI (kg/cm^2^), median (IQR)	26.8 (22.8, 30.1)	25.4 (21.5, 27.2)	0.08
EBL (mL), median (IQR)	100 (100, 150)	100 (100, 150)	0.8
Previous abdominal surgery, n (%)	34 (61)	27 (55)	0.09
ASA score ≥ 3	2 (3.6)	1 (2.2)	0.06
Hypertension, n (%)	8 (14.3)	9 (18.3)	0.05
Preoperative eGFR (mL/min), mean (standardized difference [SD])	91.7 ± 11.4	95 ± 16.5	0.9
LOS (day), median (IQR)	5.0 (4.0, 7.0)	4.0 (4.0, 5.0)	0.08
OT (min), median (IQR)	162 (135, 206)	180 (145, 230)	0.08
Clavien-Dindo			0.5
0	37 (66%)	39 (80%)	
1	4 (7.1%)	3 (6%)	
2	9 (16.1%)	6 (12%)	
3a	3 (5.4%)	0 (0%)	
3b	1 (1.8%)	0 (0%)	
4	2 (3.6%)	1 (2.0%)	
Laterality, n (%)			0.2
Right	22 (39%)	13 (27%)	
Left	34 (61%)	36 (73%)	
Surgery technique, n (%)			0.9
Radical nephrectomy VLS	45 (80%)	40 (82%)	
Simple nephrectomy VLS	11 (20%)	9 (18%)	

^1^ Kruskal—Wallis rank sum test, Fisher’s exact test, Pearson’s Chi-squared; IQR = interquartile range; BMI = body mass index; EBL = estimated blood loss; LOS = length of stay; OT = operative time; VLS = laparoscopic; ASA = American Society of Anesthesiologists.

**Table 2:** Univariable linear and logistic regression models to assess predictors for perioperative outcomes of 105 patients who underwent simple or radical laparoscopic nephrectomy at a single institution between 2018 and 2022.

	Postoperative Complications			EBL			OT			LOS		
Predictors	Odds Ratios	CI	*p*	Beta	CI	*p*	Beta	CI	*p*	Beta	CI	*p*
Experience (low vs. high)	0.50	0.20–1.19	0.1	−21	−86–44	0.5	21	1.8–40	0.04	−1.4	−3.0–0.06	0.06
BMI	0.97	0.87–1.08	0.6	11	2.9–18	0.009	3.3	1.0–5.6	0.006	−0.01	−0.19–0.18	>0.9
Age at surgery	1.03	1.00–1.08	0.08	1.3	−1.2–3.8	0.3	0.12	−0.62–0.86	0.8	0.05	0.00–0.11	0.07
Laterality (left vs. right)	0.93	0.38–2.36	0.9	−63	−131–4.8	0.072	3.6	−17–24	0.7	−1.2	−2.8–0.45	0.2

CI = confidence interval; EBL = estimated blood loss; OT = operative time; LOS = length of stay.

**Table 3:** Multivariable linear and logistic regression models to assess predictors for perioperative outcomes of 105 patients who underwent simple or radical laparoscopic nephrectomy at a single institution between 2018 and 2022.

	Postoperative Complications			EBL			OT			LOS		
Predictors	Odds Ratios	CI	*p*	Beta	CI	*p*	Beta	CI	*p*	Beta	CI	*p*
Experience (low vs. high)	0.60	0.19–1.73	0.4	−14.2	−91.8–63.3	0.7	40.5	18.7–62.3	<0.001	−0.8	−2.7–1.02	0.4
BMI	0.97	0.86–1.08	0.6	10.5	2.6–18.4	0.01	4.3	2.04–6.5	<0.001	−0.02	−0.2–0.17	0.9
Age at surgery	1.04	0.99–1.09	0.1	1.8	−1.2–4.8	0.2	0.5	−0.30–1.4	0.2	0.06	−0.01–0.13	0.08
Laterality (left vs. right)	0.93	0.33–2.71	0.9	−85.2	−161.4–9.07	0.03	12.3	−9.2–33.7	0.3	−1.3	−3.1–0.5	0.2

CI = confidence interval; EBL = estimated blood loss; OT = operative time; LOS = length of stay.

## Data Availability

All data included in this study are available upon request by contacting the corresponding author.
